# Clinical outcome of periprosthetic femoral fractures: the influence of fracture pattern and patient specific risk factors

**DOI:** 10.1007/s00068-026-03117-4

**Published:** 2026-02-23

**Authors:** Deniz D. Özman, Eftychios Bolierakis, Christian D. Weber, Rald V.M. Groven, Elizabeth R. Balmayor, Frank Hildebrand, Klemens Horst

**Affiliations:** 1 Department of Orthopedic, Trauma- and Reconstructive Surgery, RWTH Aachen University Hospital, Aachen, Germany; 2 Experimental Orthopedics and Trauma Surgery, Department of Orthopedic, Trauma- and Reconstructive Surgery, RWTH Aachen University Hospital, Aachen, Germany; 3 Department of Trauma, Hand and Reconstructive Surgery, University Hospital Muenster, Muenster, Germany

**Keywords:** Orthopaedic trauma, Periprosthetic proximal femoral fractures, Hip arthroplasty, Geriatric trauma, Osteoporosis, Nonunion

## Abstract

**Purpose:**

The incidence of periprosthetic femoral fractures (PFF) has risen, but literature on patient characteristics, risk factors, and outcomes remains limited. This study investigates radiological and clinical outcomes in relation to patient demographics and fracture patterns to identify possible risk factors and previously undetected associations.

**Methods:**

This retrospective study included patients with hip arthroplasty who sustained PFF between 2007 and 2021. Data on demographics, medical history, fracture pattern (Vancouver Classification), injury mechanism, radiological findings (e.g., osteoporosis via the Singh index), bone healing (RUSH score), bone resorption, non-union, surgical treatment, and clinical outcomes were extracted. Statistical correlations were calculated using IBM SPSS Statistics Version 28.0.0.0. (IBM Corp., Armonk, NY, USA).

**Results:**

110 patients were included, 70% female, with a mean age of 78 ± 11 years. The most common potential risk factors for PFF were uncemented stem components (69%), bone resorption (68%), female sex (70%), and osteoporosis (21%). Hospital stay was longest for B2 and B3 fractures, with B3 fractures requiring the longest intensive care unit time (mean of 3 ± 4 days, *p* = 0.041). Complications included wound infections (12%), urinary tract infections (20%) and postoperative anemia (51%). Osteoporosis was correlated with female sex and a higher risk of non-union, but did not influence bone healing.

**Conclusions:**

PFF present clinical challenges, with fracture type and complications influencing hospital stay and postoperative mobility. Type B fractures are associated with longer treatment times and higher complication rates. Osteoporosis does not appear to significantly affect bone healing in this cohort.

## Background

Due to demographic changes, the volume of total hip joint replacements has steadily risen in recent years, a trend expected to continue [1]. This increase correlates with a higher risk of periprosthetic femoral fractures (PFF), influenced by factors such as activity level, physical limitations, or medical preconditions. General risk factors for PFF, including reduced bone quality, aseptic implant loosening, bone resorption, previous revisions, and bisphosphonate use, have been described [2–4]. PFF are commonly classified using the Vancouver classification by Duncan and Masri or the Unified Classification System for Periprosthetic Fractures [5], with treatment typically involving surgical interventions such as open reduction and internal fixation, and sometimes femoral revision, depending on the fracture type, bone quality, and implant stability [6]. While Type A fractures can usually be treated conservatively and Type B2 or B3 fractures need shaft revision, type B1 and type C fractures are typically managed with plate osteosynthesis [7, 8]. Geriatric patients often have multiple medical conditions and benefit from short hospital stays, immediate physiotherapy, and early mobilization to reduce complications like decubitus ulcers, pneumonia, thrombosis, and others [9, 10]. However, early mortality rates exceeding 13% have been observed in PFF patients [11]. While the management of PFF has been extensively studied in terms of mortality rates [12], less focus has been placed on specific fracture-related risk factors and their impact on the development of complications and outcomes.

Therefore, the aim of this study was to identify factors associated with the occurrence and severity of PFF, examine the correlations between fracture patterns and these factors, and evaluate the influence of patient characteristics, fracture patterns and treatment strategies on complications and outcomes. We hypothesized that patient-related factors are associated with increased PFF severity and that certain fracture patterns, such as Vancouver-B fractures, are linked to higher complication rates and poorer functional outcomes.

## Materials and methods

This study included patients treated at a level 1 trauma center from January 2007 to December 2021 who were admitted for PFF treatment following hemi or total hip replacement. The exclusion criteria were an Injury Severity Score of > 16, an American Society of Anesthesiologists (ASA) score of > 4, and pre-fracture immobility. Data were retrospectively obtained from the hospital’s patient information system, including demographics (age, sex), body mass index, pre-existing conditions (cardiac issues, diabetes, gait disorders, use of walking aids, vision problems, history of falls, use of anticoagulants and psychotropic drugs), injury mechanism, radiological findings (fracture pattern, signs of osteoporosis, bone healing), surgical treatment, length of stay in intensive care and in the hospital, postoperative mobility, and complications (see Table 1).

**Table 1:** Postoperative complications

Infections
Wound infection Urinary tract infection Pneumonia Gastrointestinal infection
Secondary bleeding
Seroma
Acute myocardial infarction
Cardiac arrhythmia
Anemia (transfusion)
Cardiac decompensation
Electrolytic disorders
Delirium
Decubitus ulcers
Death

### Classification and treatment

**Fig. 1:**
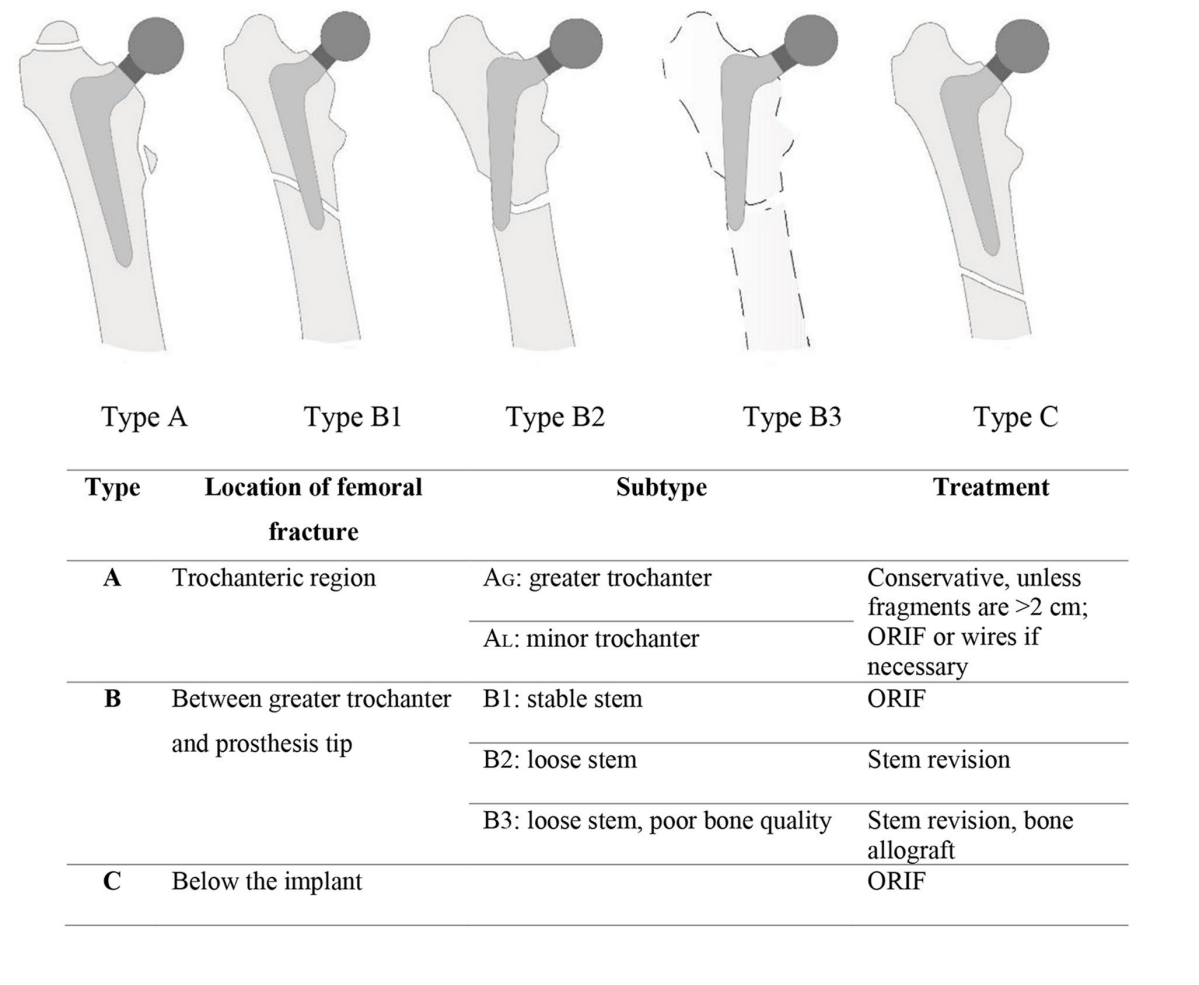
Vancouver classification of periprosthetic femoral fractures. ORIF = open reduction and internal fixation

PFF were classified using the Vancouver classification developed by Duncan and Masri (Fig. 1) [5].

### Risk factors for falls and PFF

The literature describes numerous risk factors for falls in geriatric populations, classified as internal or external [13]. To further investigate the risk of falling, the following factors were analyzed: age, sex, and pre-existing conditions such as arterial hypertension, valve problems, cardiac arrhythmia, diabetes, gait disorders, use of walking aids, vision problems, history of falls, and the use of anticoagulants and psychotropic drugs. Additionally, factors such as osteoporosis (systemic reduction of bone mass and deterioration of bone microarchitecture), bisphosphonate use, rheumatic diseases, and implant-associated aspects were evaluated, including implant loosening, bone resorption (localized bone loss due to stress shielding or physiological remodeling processes, e.g., after fracture healing), osteolysis (pathological, localized bone loss due to inflammation, tumor, or foreign body reactions) previous revisions, and whether the present implant was cemented) were evaluated.

### Osteoporosis and Singh index (SI)

The SI was determined at the trochanteric level of the contralateral hip using the method described by Hauschild et al. [14], as this area correlates more strongly with bone mineral density than the femoral neck [14]. The grading scheme was as follows:

- Grade 1: Principal compressive trabeculae are greatly reduced and no longer prominent.
- Grade 2: Only the principal compressive trabeculae are prominent, others are largely absorbed.
- Grade 3: Main tensile trabeculae are interrupted, indicating definite osteoporosis.
- Grade 4: Principal tensile trabeculae are markedly reduced but can still be traced from the lateral cortex to the upper part of the femoral neck.
- Grade 5: Principal tensile trabeculae are accentuated, Ward’s triangle appears prominent.
- Grade 6: All five trabecular groups are visible, the upper end of the femur appears completely cancellous.

### Barthel index

The Barthel index is a geriatric assessment tool used to evaluate a patient’s daily living abilities [15]. It assesses 10 activities, including bathing, grooming, eating, dressing, and mobilization. The maximum score is 100, with higher scores indicating better performance.

### Mobility at discharge

Mobility level was assessed by the physiotherapist at discharge. Patients were classified based on their ability to walk using crutches, use a rollator, require a wheelchair, or as immobile.

### Postoperative radiographs

Bone healing progress and potential non-union were determined by follow-up radiographs using the RUSH score [16, 17], as described by Chiavaras et al. [18]. The score ranges up to 30, indicating complete fracture healing [18]. A score of < 18 at 6 months postoperatively has been associated with 100% specificity (95% CI, 97%–100%) and a positive predictive value of 100% (95% CI, 73%–100%) for radiographic non-union. Follow-up was divided into four groups based on the phases of bone healing (inflammation phase, granulation phase, bony callus phase, and bone remodeling phase) [19, 20]. The final group was evaluated > 3 months postoperatively, with the last radiograph taken 357 days after surgery.

### Delayed bone healing and its risk for non-union

The term “delayed union” is used after 3 months and “non-union” after 6 months, when there is no radiographic or clinical evidence of bone healing following a fracture. Risk factors such as age, sex, comorbidities, fracture type, location, and gap width influence healing. PFF typically occur in older patients with various comorbidities, which can impair bone healing. To better assess the risk of potential non-union, the score established by Schmidmaier and Moghaddam [21] was used. This score considers factors such as fracture location, soft tissue damage, smoking, medications, and comorbidities. A score of < 10 indicates low risk, a score between 10 and 20 indicates medium risk, and a score of > 20 indicates high risk of developing non-union.

### Statistical evaluation

Data were gathered in MS Excel v16.6 (Microsoft, Redmond, WA, USA), and statistical analyses were performed using IBM SPSS Statistics Version 28.0.0.0 (IBM Corp., Armonk, NY, USA). For numerical data, normality was assessed using the Shapiro Wilk test followed by student’s t-test for independent samples or Mann-Whitney U test as appropriate. To assess correlations between variables, Pearson as well as Spearman correlation coefficients’ were used. For categorical data, the chi-square test or Fisher’s exact test was used, as appropriate. To minimize the risk of false positive findings, Bonferroni correction was applied to adjust for multiple comparisons. As a result, all reported p-values are adjusted. An alpha level of < 0.05 was considered statistically significant.

## Results

### Demographic data

From 2007 to 2021, a total of 110 patients with PFF after hip arthroplasty were analyzed. Patients’ characteristics are displayed in Table 2. The mean age of the patients was 78 ± 11 years, and most were female (70%). The mean Barthel index was 38 ± 18 points. Most patients (66%) were classified as ASA-3. Vancouver type B1 and B3 fractures were most common, accounting for 35% and 28%, respectively. The mean time from admission to surgical treatment was 3 ± 3 days, with a bimodal distribution (< 24 h = 36% and > 72 h = 35%). Three patients died during hospitalization; two due to sepsis, and one due to cardiac decompensation.

**Table 2:** Patient characteristics, data are presented as mean ± standard deviation or percentage

Characteristic	*n* = 110
Age, years	78 ± 11
Female sex	77 (70%)
Weight, kg	73 ± 23
Height, cm	162 ± 20
BMI, kg/m^2^	25.6 ± 5.8
ASA classification	
1	2 (1.8%)
2	29 (26.4%)
3	72 (65.5%)
4	7 (6.4%)
Barthel index	38 ± 18
In-hospital mortality	3 (2.7%)
Fracture type based on Vancouver classification	
A	8 (7%)
B1	38 (35%)
B2	19 (17%)
B3	31 (28%)
C	14 (13%)

BMI: body mass index. ASA: American society of anesthesiologists

### Trauma mechanism and potential reasons

Ground-level falls were the most common injury pattern (see Table 3), followed by falls from heights of < 3 m, including falls from a bed, toilet, or a few stair treads. Most accidents occurred at home, especially among older individuals, followed by those during leisure activities and incidents occurring during inpatient treatment.

**Table 3:** Trauma mechanism demographics

Characteristic	*n* = 110
Ground-level fall	74.5%
Iatrogenic	2.7%
Fall from height of > 3 m	1.8%
Fall from height of < 3 m	12.7%
Atraumatic	5.5%
Traffic accident	1.8%
Other	0.9%
Trauma location	
At home	78%
Outside	10%
While hospitalized	10%
At work	2%

Data are presented as percentage

Cardiac causes were commonly observed in patients experiencing falls in our cohort, with arterial hypertension (70%) affecting more than two-thirds of patients. This was followed by cardiac arrhythmia (25%) and valve problems (17%). Medication use, particularly anticoagulants (38%), also played a significant role, increasing the risk of more severe fracture types (B3, *p* = 0.024) while decreasing the risk of type A fractures (*p* = 0.023). External factors, such as walking aids, were used by more than half of the patients, and over one-third had a known gait disorder, followed by a positive history of falls. Diabetes (14%) and visual impairment (6%) were less frequently mentioned. Literature-based risk factors for PFF were also present in 98% of our cohort (Table 4). Notably, previously known loosening, based on radiological and clinical findings, specifically increased the risk of B3 fractures. Previous revisions and bisphosphonate use significantly increased the risk of type C fractures.

**Table 4:** Risk factors for periprosthetic femoral fractures

Characteristics	*n* = 110	Vancouver classification				
	%	A^1^	B1^1^	B2^1^	B3^1^	C^1^
Risk factors	98.2					
Osteoporosis	22.7	0.9	10.0	1.8	4.5	5.5
Polyarthritis/ rheumatism	6.4	0.9	1.8	-	3.6	-
Osteolysis	2.7	0.9	-	-	1.8	-
Loosening	18.2	0.9	-*	4.5	10.9*	1.8
Bone resorption	68.2	4.5	25.5	9.1	20.9	8.2
Previous revisions	16.4	-	6.4	0.9	4.5	4.5*
Uncemented stem	69.1	5.5	22.7	14.5	16.4	10.0
Bisphosphonates	6.4	0.0	2.7	0.0	0.0	3.6*

^1^Fisher’s exact test, *p < 0.05. Data are presented as percentage

### Complications

Postoperative complications are displayed in Table 5. The most common complications observed were postoperative anemia, remote infections such as urinary tract infections, and local wound infections. While complications occurred across all fracture types, patients with B2 fractures had a significantly higher risk of wound infections (p = < 0.05), while B3 fractures had a significantly higher incidence of urinary tract infections (p = < 0.05) compared to the other fracture types. B3 fractures also showed increased susceptibility to thrombosis/embolism, postoperative anemia, and cardiac decompensation, likely due to prolonged immobilization (see Table 11). During hospitalization, three patients died.

**Table 5:** Postoperative complications in detail

Characteristic	*n* = 110	Overall %	Vancouver classification				
			A^1^	B1^1^	B2^1^	B3^1^	C^1^
Local							
Wound infection		11.8	-	1.8	4.5*	5.5	-
Bleeding		4.5	-	0.9	0.9	1.8	0.9
Seroma		2.7	-	0.9	0.9	0.9	-
Remote							
UTI		20	0.9	1.8*	4.5	10.0*	2.7
Pneumonia		12.7	0.0	2.7	3.6	4.5	1.8
Gastrointestinal		7.3	0.0	1.8	2.7	1.8	0.9
Other							
Thrombosis/embolism		2.7	-	-	-	2.7*	-
AMI		2.7	-	0.9	0.9	0.9	-
Cardiac arrhythmia		7.3	-	0.9	1.8	3.6	0.9
Anemia (transfusion)		50.9	0.9*	13.2	13.2*	**19.8**	5.7
*Unknown*	4						
Decompensation		16.4	-	2.7	0.9	10.0*	2.7
Electrolyte disorder		4.5	0.9	0.9	0.9	-	1.8
Delirium		9.1	-	1.8	0.9	4.5	1.8
Decubitus ulcers		5.5	-	1.8	1.8	1.8	-

^**1**^Fisher’s exact test, *p < 0.05. Data are presented as percentage. UTI: urinary tract infection. AMI: acute myocardial infarction

### Osteoporosis

The SI was evaluated for 52 patients (see Table 6). Because the score cannot be assessed in patients with bilateral hip prostheses, 54 patients were excluded. Four patients had missing radiologic images (anteroposterior pelvis). Normal SI values between 4 and 6 were found in 19.0% of patients, while values of ≤ 3 were found in 28.2% of patients.

**Table 6:** SI determined from preoperative radiographs in ipsilateral trochanteric region

Characteristic	*n* = 110	%
SI	52	47.3
Unknown	58	52.7
1		2.7
2		8.2
3		17.3
4		3.6
5		4.5
6		10.9
Osteoporosis (determined from SI ≤ 3)		28.2
Osteoporosis overall		45.5

Data are presented as percentage. SI: Singh index

### Bone healing in postoperative radiographs

Patients with osteoporosis theoretically have a higher risk of developing non-union according to the Schmidmaier and Moghaddam score [21] (see Table 7). However, no significant difference was observed in the duration of bone healing between the two patient groups. Bone healing also occurred at the same rate in both groups over time (see Table 8).

**Table 7:** Osteoporosis and its relation to risk of non-union

Characteristic	Non-union risk score according to Schmidmaier and Moghaddam [21]	*p*-value^1^
Osteoporosis	12 ± 7	0.018*
No osteoporosis	8 ± 5	

^**1**^Independent t-Test, *p < 0.05. Data are presented as mean ± standard deviation

**Table 8:** Bone healing based on the RUSH score

Characteristic	RUSH score 1 1–14 days	*p*-value^1^	RUSH score 2 15–42 days	*p*-value^1^	RUSH score 3 43–91 days	*p*-value^1^	RUSH score 4 > 3 months	*p*-value^1^
n	102		47		38		26	
Osteoporosis	11 ± 2	0.96	13 ± 4	0.23	16 ± 6	0.43	23 ± 8	0.68
No osteoporosis	11 ± 2		15 ± 4		18 ± 6		24 ± 6	

^**1**^Independent t-Test, *p < 0.05. Data are presented as mean ± standard deviation

### Association of different fracture patterns with clinical course and outcome

The mean time from admission to surgical treatment was 3 ± 3 days, with type C fractures being treated the fastest (see Table 9). The overall hospital stay was longest for patients with B2/3 fractures. B3 fractures had the longest duration in the intensive care unit (ICU) among all fracture types.

**Table 9:** Overall stay and outcome of the different fracture patterns

Characteristics	Time from admission to surgery	*p*-value^1^	In-hospital stay	*p*-value^1^	ICU stay	*p*-value^1^
**Days**	3 ± 3		19 ± 14		2 ± 3	
**Vancouver-Classification**						
**A** **r** ^**1**^	3 ± 6 −0.17	0.07	10 ± 6 **−0.21**	0.025*	1 ± 1 −0.11	0.26
**B1** **r** ^**1**^	3 ± 2 −0.1	0.34	16 ± 8 −0.11	0.25	1 ± 3 **−0.22**	0.019*
**B2** **r** ^**1**^	5 ± 4 **0.24**	0.01*	23 ± 15 0.17	0.08	1 ± 1 0.1	0.36
**B3** **r** ^**1**^	4 ± 3 **0.2**	0.03*	24 ± 20 **0.2**	0.04*	3 ± 4 **0.3**	0.001*
**C** **r** ^**1**^	1 ± 1 **−0.3**	0.002*	14 ± 6 −0.12	0.22	2 ± 4 −0.1	0.3

^1^Spearman correlation, *p < 0.05. Data are presented as mean ± standard deviation

### Factors influencing postoperative intensive care and the risk of complications

The need for intensive care was influenced by several factors (see Table 10). Remote infections and cardiac diseases were central contributors. Conversely, longer ICU stays significantly increased the risk of infectious complications, particularly urinary tract infections and pneumonia. Regarding fracture type, B3 fractures had a significantly higher risk of requiring intensive care, while B1 fractures had a lower risk.

**Table 10:** Need for intensive care treatment

ASA classification	*n* = 110 %	*r*^1^ (*p*-value)
1	1.8	
2	10	−0.2 (0.03)*
3	37.3	
4	6.4	0.2 (0.014)*
Local infections	10.9	0.16 (0.1)
Remote infections	27.3	0.4 (< 0.001)*
UTI	16.4	0.26 (0.005)*
Pneumonia	11.8	0.3 (0.002)*
Gastrointestinal infections	6.4	0.18 (0.06)
Other		
Cardiac arrythmia	7.3	0.25 (0.008)*
Thrombosis/embolism	1.8	0.03 (0.7)
AMI	2.7	0.15 (0.1)
Arterial hypertension	43.6	0.21 (0.027)*
Anticoagulation	29.1	0.33 (< 0.001)*
Anemia	36.8	0.28 (0.04)*
Decompensation	13.6	0.25 (0.009)*
Electrolyte disorder	3.6	0.1 (0.26)
Delirium	7.3	0.15 (0.1)
Gait disorders	20.9	0.2 (0.05)*

^1^Pearson correlation, *p < 0.05. Data are presented as percentage. ASA: American society of Anesthesiologists. UTI: urinary tract infection. AMI: acute myocardial infarction

### Patient mobility and discharge outcomes

Longer ICU stays were associated with a longer overall hospital stay (*r* = 0.36, *p* = 0.001) and had a major influence on the mobility status at discharge. Patients admitted to the ICU had a significantly higher risk of being discharged in a wheelchair (*p* < 0.001) and a significantly lower chance of being discharged with walking sticks (*p* = 0.005). This trend was particularly evident in B3 fractures (see Table 11), where the risk of being discharged in a wheelchair was significantly higher. In contrast, patients with type A fractures had a lower risk of reduced mobility by wheelchair and a significantly higher likelihood of being mobilized with walking sticks.

**Table 11:** Mobilization upon discharge

Characteristics	Mobilization upon discharge	Walking stick	*p*-value^1^	Rollator	*p*-value^1^	Wheel- chair	*p*-value^1^	Immobile	*p*-value^1^
**Vancouver classification**									
**A** **r** ^**1**^		3.6% **0.24**	0.01*	3.6% 0.04	0.7	- **−0.2**	0.04*	- −0.4	0.7
**B1** **r** ^**1**^		7.3% 0.7	0.45	15.5% 0.003	0.97	9.1% −0.14	0.24	1.8% 0.18	0.05
**B2** **r** ^**1**^		1.8% −0.1	0.4	9.1% 0.1	0.4	6.4% 0.04	0.67	- −0.6	0.52
**B3** **r** ^**1**^		1.8% −0.2	0.06	10.9% −0.06	0.52	13.6% **0.21**	0.03*	- −0.08	0.4
**C** **r** ^**1**^		2.7% 0.04	0.6	5.5% −0.006	0.95	4.5% 0.024	0.8	- −0.05	0.6

^1^Spearman correlation, *p < 0.05. Data are presented as percentage

## Discussion

The incidence of PFF is increasing, presenting challenges in medical management and surgical therapy. Existing literature on PFF is sparse regarding patient demographics, risk factors, complications, and clinical outcomes. Therefore, this study aimed to address these gaps. The results can be summarized as follows:

1. Ground-level falls are the most common cause of PFF in the elderly, mostly occurring at home. Cardiac issues and anticoagulant use are primary contributors. Known risk factors were observed in nearly all patients, including a history of revision surgery, bone resorption, and osteoporosis, which were associated with PFF severity.
2. Vancouver-B fractures represent the majority of fracture types, significantly associated with complications, including local and remote infections, ICU treatment, longer overall hospital stay, and reduced mobility at discharge.
3. ICU treatment is influenced by ASA status, infections, and comorbidities, leading to prolonged hospital stays and reduced mobility at discharge.
4. Although osteoporosis is generally associated with a higher risk of non-union, it did not appear to influence bone healing in patients without healing disturbances.

### Demographic data

The presented cohort aligns with other populations previously studied regarding PFF [12, 22]. In addition to higher age, a predominance of female patients is commonly found in studies focusing on PFF. Most patients in our cohort were classified as ASA grade 2 or 3, consistent with findings by El Khassawna et al. [12]. Although these patients remain engaged in daily activities, sports, and social events, pre-existing factors such as age, osteoporosis, and prosthetic status may contribute to PFF occurrence and clinical progression, as noted by Patsiogiannis et al. [23]. Most fractures were classified as type B, which is consistent with other studies and explained by Lindahl et al. [24], who attributed this allocation to the high incidence of prosthesis loosening over time, prosthesis design, the potential for revision surgery, and regular follow-up and early detection. Therefore, the study population can be considered representative of the results discussed below.

### Risk factors for the development of PFF

Consistent with Fuller [25], ground-level falls were the main cause of PFF in the elderly, particularly occurring at home, aligning with Sartini et al. [26]. Although not investigated in this study, previous research [13, 27] identified potential in-home fall risks, including inadequate lighting, slippery floors and tripping hazards. Higher age often correlates with cardiovascular comorbidities and anticoagulant use. Both are significant risk factors for fractures of all types, including hip fractures, as shown by Huang et al. [28]. Anticoagulants were found to reduce bone mineralization, as described by Signorelli et al. [29], which explains the significant association between anticoagulation therapy and type B3 fractures in the present study. These fractures are characterized by poor bone quality and a loose implant. Additionally, multiple hip revisions or osteoporosis lead to reduced bone stock and poor bone quality, which were confirmed as risk factors for PFF in this cohort. The impact of osteoporosis on bone healing and non-union remains controversial [30], and possible correlations have not yet been well described in patients with PFF [31, 32]. However, several studies offering conflicting findings in osteoporosis and bone healing [33–37]. Although a higher risk for non-union was found in our population with osteoporosis, osteoporosis did not influence healing duration in patients without healing disturbances. Osteoporotic therapy with bisphosphonates, which are discussed as an independent risk factor for PFF [4], was positively associated with the occurrence of PFF in our population, particularly in type C fractures. Furthermore, uncemented stem components were found to be a potential risk factor for PFF [2], which was observed in 11 of 14 patients with type C fractures (10% of all PFFs in our cohort). Reduced bone mineralization increases brittleness, raising the risk of microscopic cracks over time [38]. Although the above findings align with current literature supporting the development of atypical femur fractures triggered by the reduction in bone mineral density in the geriatric population and the long-term use of bisphosphonates in the elderly [39], Karam et al. [40] could not prove an association between the fracture classification and whether the stem was cemented. This observation may be due to significant differences in the compared patient groups, as cemented patients were older and had a shorter interval between prosthesis implantation and fracture incidence, and varus stem position was significantly higher. Moreover, osteoporosis was not taken into account. Ettinger et al. [38] discussed reduced collagen plasticity and fragility of the bone as causes of atypical femur fractures in the elderly, while Klasan et al. [41] demonstrated a 25% higher load to failure in cemented stems. Thus, it may be assumed that in combination with advanced osteoporosis and weakened bone, uneven force distribution - potentially occurring with uncemented stem components - could lead to the formation of stress risers beneath the prosthesis. This proposed mechanism remains speculative and was not directly evaluated in the present study. While literature regarding PFFs and specific fracture patterns remains sparse, this study supports previous findings linking reduced bone mineralization, anticoagulants and bisphosphonate use with the occurrence of such specific fracture patterns. Further molecular, histologic, and biomechanical research is needed to better understand the interactions between age-related factors, comorbidities, and medication effects on bone mineralization, ultimately improving surgical approaches in PFF treatment.

### Further clinical course and mobility

The data regarding the time from diagnosis to definitive treatment presented by the Compose Study team were confirmed [42]. Type C fractures were treated fastest, while type B2/B3 fractures had the longest time to surgery due to their complexity and treatment protocols, with type C fractures generally requiring open/closed reduction and internal fixation, while B2/B3 fractures often necessitate complex revision surgery with implant replacement. This was also reflected in longer hospital stays for B2/B3 fractures, consistent with Lyons et al. [43], who reported longer stays for these fractures compared to type C. In our study, the majority of B2 fractures (17 out of 19 cases) were treated with revision arthroplasty. One fracture was treated with plate osteosynthesis and another with cerclage alone. A recent study by Wilke et al. has shown that selected B2 fractures can also be successfully treated with plate osteosynthesis [44]. This suggests that alternative strategies to our predominantly revision arthroplasty-based approach could improve patient outcomes and should be reconsidered. In this study, patients with B3 fractures also had the longest ICU stays, which is attributable to the need for major revision surgery. The ICU stay was significantly correlated with a higher risk of remote infections, blood loss, thrombosis/embolism and cardiac decompensation [45]. Moreover, a prolonged ICU and hospital stay was associated with reduced mobility at discharge, likely due to loss of muscle strength and endurance, and its effects on mental state and motivation [9, 46]. Accordingly, B3 fractures had a higher risk of being discharged in a wheelchair compared to type A fractures in our study population. Given our findings and the prolonged ICU and hospital stays, particularly for B3 fractures, strategies to minimize ICU admission and promote early mobilization are especially important. Careful perioperative optimization of comorbidities - especially cardiac disease - through early risk stratification, medication management, and multidisciplinary planning can reduce perioperative complications and ICU need. This could be achieved through the implementation or adaptation of standardized perioperative care protocols tailored to high-risk patients with PFF. Such protocols may help streamline preoperative assessment, optimize the management of comorbidities, and support early postoperative mobilization, as demonstrated by Bernstein et al. in an arthroplasty patient cohort [47]. Anesthesia optimization, including the use of regional techniques when appropriate, may further decrease ICU requirements. Effective pain management, patient education, and appropriate use of mobility aids are critical to facilitate early mobilization. As literature on PFF in this context remains sparse, further research is needed to optimize perioperative management and to ensure prompt mobilization after surgery.

### Outcome and mortality

Mortality rates for patients with PFF during hospitalization vary widely in the literature, with an average of 2.4% reported by Lamb et al. [11]. Mortality within 30 days (3.3%) and 1 year (13%) also show variability. In our cohort, three patients died of infection or cardiac conditions during their hospital stay, which corresponds to a rate of 2.7%, consistent with the results mentioned above. Although further follow-up after discharge was not possible because of data protection laws, the pooled 30-day mortality rate for PFF was found to be comparable to that for proximal femoral neck fractures [48]. All patients who died in our study sustained a type B fracture. Compared to patients with type A and C fractures, those with B1 and B3 fractures had both a longer time from hospital admission to surgery and a longer overall hospital stay. Specifically, patients with B1 fractures were more likely to have pre-existing cardiac conditions, which may have contributed to delayed surgery. According to the German Federal Joint Committee guidelines, proximal femoral fractures must be treated within 24 h to reduce complications and improve mortality. No in-hospital deaths occurred among patients with type C fractures, and no significant complications were observed, with a mean time to treatment of 1 ± 1 days. In consideration of our findings and a closer examination of clinical outcomes, particularly for type C fractures, surgical treatment of all fracture types - whether for proximal femoral fractures or PFF - within 24 h may be advisable, with particular focus on type B fractures, as these often account for the majority of periprosthetic femoral fractures [24]. Early surgical intervention could further improve patient outcomes and, importantly, reduce potential mortality. This recommendation aligns with the findings of Wulbrand et al. [49], who highlighted that patients with periprosthetic femoral fractures benefit from undergoing surgery within 24 h.

### Strengths and limitations

The strengths of this study include the extensive data available from the hospital system and its focus on risk factors for an injury pattern that is becoming increasingly important in older patients. It also highlights the role of medical preconditions and the postoperative clinical course. However, the study has limitations, including its retrospective design, the absence of molecular, histologic, and biomechanical data, and its conduct at a single-center institution. Due to the limited sample size and substantial intercorrelation between several variables, the study design and data structure did not permit multivariate regression analyzes. As a result, only univariate analyses were performed, which limits the ability to identify independent predictors. Further research with larger cohorts and more comprehensive analyses are necessary to investigate and build upon our findings. One additional limitation is the continued use of uncemented femoral stems in our institution, particularly in patients with presumed good bone quality. While this reflects common clinical practice, uncemented stems have been identified as a potential risk factor for PFF in the current literature. Future studies should consider a more detailed risk assessment regarding implant selection, especially in light of evolving evidence on implant-related fracture risk. In this context, and given the limited data availability until 2021 and the importance of an interdisciplinary approach to improving clinical care, especially for geriatric patients, future research should extend these analyzes to more recent patient cohorts.

## Conclusion

The results of this study demonstrate that patients with medical preconditions, especially those with a cardiovascular history, anticoagulant therapy, and poor bone quality, are more likely to sustain PFF. Complex fractures were associated with a higher complication rates, prolonged hospitalization, and reduced mobility at discharge. Although osteoporosis was associated with a higher risk of non-union in general, bone healing was not affected. Thus, prompt surgical intervention, early identification of risk factors, and awareness of potential complications in treating these complex cases can significantly improve patient care.

## Data Availability

The data for this publication will be made available upon reasonable request.

## Abbreviations

PFF: Periprosthetic femoral fractures
ASA: American Society of Anesthesiologists
